# Predictors of person-centered maternity care: the role of socioeconomic status, empowerment, and facility type

**DOI:** 10.1186/s12913-018-3183-x

**Published:** 2018-05-11

**Authors:** Patience A. Afulani, Takudzwa S. Sayi, Dominic Montagu

**Affiliations:** 1 University of California, San Francisco, USA; 2 University of South Florida, Tampa, USA

**Keywords:** Person-centered care, Maternity care, Respectful care, Kenya, Sub-saharan Africa, Socioecononmic status, Empowerment, Facility-based delivery

## Abstract

**Background:**

Low use of maternal health services, as well as poor quality care, contribute to the high maternal mortality in sub-Saharan Africa (SSA). In particular, poor person-centered maternity care (PCMC), which captures user experience, contributes both directly to pregnancy outcomes and indirectly through decreased demand for services. While many studies have examined disparities in use of maternal health services, few have examined disparities in quality of care, and none to our knowledge has empirically examined disparities in PCMC in SSA. The aim of this study is to examine factors associated with PCMC, particularly the role of household wealth, personal empowerment, and type of facility.

**Methods:**

Data are from a survey conducted in western Kenya in 2016, with women aged 15 to 49 years who delivered in the 9 weeks preceding the survey (*N* = 877). PCMC is operationalized as a summative score based on responses to 30 items in the PCMC scale capturing dignity and respect, communication and autonomy, and supportive care.

**Results:**

We find that net of other factors; wealthier, employed, literate, and married women report higher PCMC than poorer, unemployed, illiterate, and unmarried women respectively. Also, women who have experienced domestic violence report lower PCMC than those who have never experienced domestic violence. In addition, women who delivered in health centers and private facilities reported higher PCMC than those who delivered in public hospitals. The effect of employment and facility type is conditional on wealth, and is strongest for the poorest women. Poor women who are unemployed and poor women who deliver in higher-level facilities receive the lowest quality PCMC.

**Conclusions:**

The findings imply the most disadvantaged women receive the lowest quality PCMC, especially when they seek care in higher-level facilities. Interventions to reduce disparities in PCMC are essential to improve maternal outcomes among disadvantaged groups.

**Electronic supplementary material:**

The online version of this article (10.1186/s12913-018-3183-x) contains supplementary material, which is available to authorized users.

## Background

Maternal mortality has fallen by almost 50% since 1990, yet it is still 14 times higher in developing than developed regions. Of the estimated 800 pregnancy-related deaths occurring daily, about 99% occur in low- and middle-income countries, with about two-thirds occurring in sub-Saharan Africa (SSA) alone [1]. In 2015, the estimated maternal mortality ratio (per 100,000 live births) for SSA was at a high 546—with that of Kenya at 510—compared to about 12 in high-income regions [2]. Reaching the Sustainable Development Goal (SDG) target of reducing the global maternal mortality ratio to less than 70 is unlikely, given such high mortality to begin with. Additionally, maternal mortality is higher among young adolescents and poor and uneducated women [1, 3] and goes hand-in-hand with maternal morbidities and neonatal mortality. Two factors contribute to these poor outcomes: low use of maternal health services and poor quality care [4–7].

About three-quarters of maternal deaths are due to complications during labor, birth, and the first 24 h postpartum [4]. These complications are difficult to predict, but can be effectively managed and deaths averted if recognized and treated promptly [8, 9]. Skilled attendance at birth is therefore important to reduce maternal mortality. In most of SSA, skilled attendance only occurs in health facilities. Yet only about half of births in SSA occur in health facilities [4–7]—with wide disparities by socioeconomic status (SES) [10, 11]. For example, the 2014 Kenya Demographic and Health Survey (DHS) showed that 61% of women with a live birth in the five years preceding the survey gave birth in a health facility. But only about 25% of women with no education and 31% of women in the lowest wealth quintile gave birth in health facilities. On the other hand, among women with secondary or higher education and those in the highest wealth quintile, 85 and 93% respectively gave birth in health facilities [12].

Poor quality care contributes directly to high maternal mortality in SSA through poor identification and management of pregnancy complications; and indirectly through decreased demand for services [13, 14]. Person-centered care, which captures user experience and is best judged by users, in particular, influences health-seeking behavior. Person-centered maternity care (PCMC) is maternity care that is respectful of and responsive to women’s preferences, needs, and values [15, 16]. It includes system and provider responsiveness, patient-provider communication, interpersonal treatment, patient engagement, and related constructs [16–19]. Disrespectful, abusive, and neglectful treatment of women in facilities during childbirth indicates very poor PCMC. When women share these poor experience in their communities, it leads to poor community perceptions of quality of care, which deters other women from giving birth in health facilities [13, 20–24].

Several studies have examined disparities in use of maternal health services, but few have empirically examined disparities in quality of maternal health care in SSA. Disparities in PCMC are however thought to be contributing to disparities in use of skilled birth attendants, and in maternal and neonatal outcomes [25]. Most quality of maternal health care data have been collected at the facility level, precluding individual–level analyses [26–30]. These studies, including in Kenya, however show that quality of antenatal, delivery, and postnatal care is poorer in poor communities [31–33]. The few studies that have examined quality of care at the individual level (using antenatal services received) also show SES and facility-based differences in quality of care [34, 35]. Studies on women’s experiences during childbirth also suggest poor and younger women are more likely to be mistreated and stigmatized in health facilities [21]. These are, however, mostly qualitative studies with small sample sizes, which preclude objective evaluation of SES differences. Fewer studies have thus systematically examined disparities in PCMC in SSA. This dearth of quantitative studies may be due to lack of validated tools to measure PCMC. In this study, we use a recently developed PCMC scale [16]. This study is among the first to quantitatively examine predictors of PCMC in SSA.

This study aims to examine factors associated with PCMC, particularly the role of household SES, personal empowerment, and type of delivery facility. Drawing on prior work, we hypothesize that higher SES will be associated with higher PCMC. Potential reasons for this relationship include the following: Women with higher SES may live in areas where quality of care is higher or they can physically access and afford high quality care. They may also have higher expectations of care and the ability to advocate for better care; and may be more likely to have relationships with health personnel, which facilitate receipt of high quality services [34, 36–41]. For PCMC in particular, the narrower social power gap between high SES women and health personnel may cause health providers to be more respectful and supportive than might otherwise be the case [36, 42]. While mistreatment of poor women may be intentional in some circumstances, we posit that this is often not the case. As Leape noted, disrespectful behavior towards patients thrives in a culture that tolerates and supports disrespect, and individual biases may reinforce patterns of abuse [43, 44]. Thus, in hierarchical societies where people of low status are more likely to be disrespected, providers may be unconsciously mistreating women of low social status.

We also hypothesize that a woman’s personal empowerment will be associated with PCMC. The rationale is that women who are personally empowered (economically, cognitively, and socioculturally) may be able to overcome barriers to receiving good quality care [45]. Kabeer defines women’s empowerment as: “the expansion of people’s ability to make strategic life choices in a context where this ability was previously denied to them” [46]. In a setting where PCMC is low, empowerment increases one’s ability to demand good care. Empowerment incorporates three inter-related components: resources (access to material, human, and social resources); agency (processes of decision making and negotiation); and achievements (well-being outcomes) [46]. Resources and agency increases a woman’s ability to access and pay for and to demand or negotiate for good quality care [45].

We also examine other key determinants of quality of care net of characteristics of the recipients of care. These include characteristics of the facility and providers. Differences in quality of maternal health care by facility tend to depend on the dimensions of quality one is examining. Some studies find higher technical quality in higher-level public facilities, while others find higher quality interpersonal quality in private facilities [27, 34, 37]. Since PCMC captures the interpersonal quality, we hypothesize that PCMC will be lower in higher-level public facilities than lower-level public facilities and private facilities. In busy and crowded higher-level facilities with a correspondingly high ratio of patients to skilled providers, PCMC might be lower due to the stressful working environment. Individual characteristics of providers are also important as disrespectful behavior towards patients may stem from characteristics of providers and their responses to stressful environments [43, 44].

The predictors of interest may interact in ways that affect care, thus we use mediation and moderation analyses to explore the questions: How much of the effect of household wealth is accounted for by women’s personal empowerment? Is the effect of empowerment conditional on wealth? How much of the SES effect is due to the type of facility in which care is received? Is the SES effect conditional on the type of facility?

## Methods

### Setting

Data for this analysis are from a larger study on perceived quality of maternity care in a rural county in western Kenya [16, 17]. The study county, which is divided into 8 sub-counties, has a population of approximately one million [47]. Close to half of the study county population (43%) lives below the poverty line and very few women of reproductive age (3%) have more than a secondary education. The study county has an estimated 40,000 annual births, and women aged 15–19 years make up about a quarter of women of childbearing ages [12, 47]. About half of women in the study county give birth in health facilities [12]. There is one referral hospital in the county, along with seven sub-county hospitals, 18 health centers, several dispensaries, and a few faith-based and private health facilities. The health care provider/population ratio is about 32, 19, and 4 nurses, clinical officers, and doctors, respectively, per 100,000 people in the county [48].

### Data collection

The data are from a survey conducted in August and September 2016, with women aged 15–49 years who delivered in the nine weeks preceding the survey. The data collection is described in detail elsewhere [16]. Women were recruited using a multistage sampling approach. In the first satge, we divided the county into eight strata based on the eight sub-counties. Next we randomly selected ten community health units within each sub-county. (Community health units are the lowest level in the health service delivery structure covering a defined geographic area set so as to include approximately 5000 people. Community health extension workers and community health volunteers are assigned to each unit where they offer promotive, preventative, and basic curative health services [49]). A community health volunteer assisted in identifying women who delivered in the 9 weeks preceding the study within each of the 10 units that were randomly selected. Twelve trained data collectors conducted the interviews in English, Swahili, and Luo in private spaces in health facilities or in the homes of the respondents. About 1000 women were interviewed, with response rate above 98%. We use data from women who delivered in a health facility (894) and who had complete information on all the relevant variables for this analysis (*N* = 877). All participants provided written informed consent after receiving information about the study. They were given an incentive of 200 Kenyan shillings (~$2). Ethical approval for the study was provided by the institutions listed in the ethics statement.

### Measures

#### Dependent variable (outcome): Person-centered maternity care (PCMC) score

The focal dependent variable is the PCMC score—a summative score from responses to items in the PCMC scale. The scale is made up of 30 items measuring multiple domains of PCMC, including dignity and respect, communication and autonomy, and supportive care. Each item is on a 4-point response scale—0: “no, never,” 1: “yes, a few times,” 2: “yes, most of the time,” and 3: “yes, all the time.” The minimum possible score on the scale is therefore 0 and maximum possible score is 90, with a lower score implying poorer PCMC. The full list of items and distribution of the items are shown in Additional file 1. The scale has good internal consistency reliability, with Cronbach’s alpha of 0.88; and high content, construct, and criterion validity. The scale was developed following literature reviews to generate items, expert reviews to assess content validity, and cognitive interviews to assess respondent understanding of questions and their relevance to them. Iterative revisions were done at each stage. The final items were then administered in two surveys in Kenya and the data used for the psychometric analysis. The dataset for this paper is one of the two datasets used for the validation of the scale. The development and validation of this tool in Kenya is described in detail elsewhere [16].

#### Independent variables (predictors)

We identified five sets of factors that might affect the quality of PCMC a woman receives: socioeconomic factors, facility and provider characteristics, women’s health status, familiarity with the health system, and demographic factors. The focal independent variables were the socioeconomic factors, which capture a woman’s SES and her personal empowerment. SES refers to the social rank of an individual and her family, and incorporates economic status usually measured by income and/or wealth and social status usually measured by education and/or occupation [50]. We operationalize SES in this analysis as: *Household wealth* (measured in quintiles, calculated from a wealth index based on 13 questions on household assets) [51], *Education, Occupation*, and *Partners education and occupation*.

We include the following variables capturing various aspects of women’s empowerment: *Employment status*: a survey question asking, “Do you do any work for which you are paid?” This captures economic empowerment—access to and control over the means to make a living, and receiving the material benefits of this access [52]. Access to work and income increases economic independence and therefore independence overall [53]. *Literacy:* Two questions on whether one can read and write, with responses as “no,” “yes, with some difficulty” and “yes, very well.” Literacy and also education are potential measures of cognitive and psychological empowerment, which includes knowledge about rights, self-esteem, and self-efficacy [53, 54]. We also included two composite measures on *participation in household decision-making* and *attitudes towards domestic violence* (from questions in the DHS module on empowerment shown in Additional file 2) to measure sociocultural empowerment, which captures gender norms, including norms against gender-based violence [55]. The responses to the individual questions were recoded and summed to create scores, which were then dichotomized at the median to create the empowerment categories. In addition we included experience of domestic violence as a predictor, as disrespect and abuse of women is thought to be linked to gender-based violence [56].

For facility and provider characteristics, we included three variables: *Facility type*: the facility the woman delivered in, grouped into public/government hospital (higher-level), health center (lower-level), and private/mission health facility (too few to group by levels). *Provider type:* whether the delivery was assisted by a doctor, clinical officer, nurse or midwife, a combination of these providers, or an unskilled attendant (neither doctor, clinical officer, nurse or midwife). *Provider sex:* whether the delivery providers were male, female, or included both males and females.

To account for the effect of women’s health status on how they are treated, we included variables on whether they had any pregnancy and delivery complications and their assessment of their severity. For familiarity with the health system, we included a variable on whether they had previously delivered in a health facility and the timing and frequency of antenatal care. We also included demographic factors such as age, marital status, parity, tribe, and religion that might affect how one is treated in a facility. Finally, we controlled for the timing and setting of the interview which might affect their responses.

### Analysis

Initial analyses involved descriptive statistics for the sample – means for continuous variables and proportions for categorical variables. Next we examined the bivariate associations between the independent variables and the dependent variable through cross tabulations of the mean PCMC scores by the various predictors [57–59]. We also fitted unadjusted ordinary least squares (OLS) regressions, as the PCMC score is a normally distributed continuous variable [60]. We used multivariable OLS models to examine predictors of PCMC net of other factors. We built the multivariable models starting with household wealth and sequentially added other predictors that were significant in the bivariate models. We combined wealth into three categories in the multivariable analysis to allow comparison between the richest and poorest women. We conducted post estimation tests to check if additional variable(s) improved the model, and assessed model fit. We also conducted collinearity tests to exclude variables that were closely correlated. Only variables that improved the model and were not collinear with other items in the model are included in the final multivariable model. We conducted sensitivity analysis using a binary measure of PCMC, the PCMC sub-scales, and selected items in the PCMC scale.

We conducted two sets of additional analysis. The first was mediation analysis to assess how much of the household wealth difference in PCMC was accounted for by a woman’s personal economic empowerment (and also the reverse); and how much of the SES difference was accounted for by the facility and provider characteristics. We used nested models with the difference of coefficients (c-c’) method for the mediation—where the mediated effect is the difference between the coefficients of the focal independent variable in the model without the mediator (total effect = c) and the model with the mediator (direct effect = c’). That is, *mediated* or *indirect effect* = *total effect* minus *direct effect* (c-c’); and *proportion of total effect mediated* = *mediated/total effect* = (c-c’)/c = 1 – c’/c) [61, 62]. The second was moderation analysis to determine whether the effects of employment status and facility type are conditional on wealth. Here we included two interaction terms for employment status and wealth and facility type and wealth.

## Results

### Descriptive

Table 1 shows the univariate and bivariate distributions of the study variables. The average age is about 25 years, with about 19% being less than 20 years old. Approximately 78% are married, with average parity of three; 27% have more than four children. About 56% have only primary education or less and 25% are employed. About 67% belong to the dominant tribe of the County—Luo; and almost all are Christians. About two-thirds had more than 4 antenatal care visits. Close to nine in ten women delivered in a public health facility, with 46% in public hospitals and 41% in health centers. About 13% delivered in private facilities. About 61% had previously delivered in a health facility. Close to 60% of the interviews occurred outside a health facility. The average postpartum length of respondents is 5 weeks with a range of zero to nine weeks. The average PCMC score is about 59 (SD = 14.0 l; range = 21–90).

**Table 1:** Univariate and Bivariate Distribution of study variables

	Univariate		Bivariate statistics: PCMC scores by predictors				
			Crosstabs		OLS regression		
	*No.*	*%*	*Mean*	*SD*	*Coeff.*	*CI*	
Total N	877	100.0	59.0	14.0			
Age							
15 to 19 years	162	18.5	57.1	14.5	0.0	[0	0]
20 to 29 years	511	58.3	59.4	14.0	2.3	[−0.17	4.78]
30 to 48 years	204	23.3	59.2	13.5	2.1	[−0.84	4.94]
Marital status							
Single	140	16.0	56.4	14.6	0.0	[0	0]
Partnered/Cohabiting	3	0.3	55.3	13.3	−1.1	[−17.0	14.9]
Married	687	78.3	59.7	13.8	3.3*	[0.76	5.84]
Widowed	35	4.0	55.4	15.3	−1.0	[−6.14	4.22]
Divorced/Separated	12	1.4	58.1	14.0	1.7	[−6.55	9.94]
Number of births							
1.0	290	33.1	58.6	14.9	0.0	[0	0]
2.0	185	21.1	60.1	14.1	1.6	[−1.02	4.15]
3.0	163	18.6	60.0	12.7	1.4	[−1.27	4.10]
4 or more	239	27.3	57.8	13.6	−0.8	[−3.18	1.62]
Education							
No school/Primary	495	56.4	58.1	14.1	0.0	[0	0]
Post-primary/Vocational/Secondary	271	30.9	59.2	13.9	1.1	[−0.94	3.20]
College or above	111	12.7	62.1	13.2	4.0**	[1.12	6.87]
Literacy: writing							
No, cannot write	31	3.5	53.5	14.7	0.0	[0	0]
Yes, with some difficulty	141	16.1	57.6	13.0	4.1	[−1.32	9.55]
Yes, very well	705	80.4	59.5	14.1	6.0*	[0.99	11.0]
Literacy: reading							
No, cannot read	37	4.2	53.6	13.2	0.0	[0	0]
Yes, with some difficulty	129	14.7	57.9	13.1	4.3	[−0.79	9.44]
Yes, very well	711	81.1	59.4	14.2	5.8*	[1.17	10.4]
Employed							
No	658	75.0	57.4	13.6	0.0	[0	0]
Yes	219	25.0	63.7	14.1	6.4***	[4.29	8.50]
Work or relation in health facility							
No	819	93.4	58.8	14.0	0.0	[0	0]
Yes	58	6.6	61.3	14.3	2.5	[−1.19	6.28]
Wealth Quintile							
Poorest	190	21.7	56.4	14.1	0.0	[0	0]
Poorer	190	21.7	57.8	13.5	1.4	[−1.44	4.16]
Middle	135	15.4	58.6	14.5	2.2	[−0.92	5.23]
Richer	172	19.6	61.7	13.4	5.3***	[2.42	8.17]
Richest	190	21.7	60.4	14.1	3.9**	[1.13	6.73]
Wealth Quintile							
Poorest	380	43.3	57.1	13.8	0.0	[0	0]
Middle	135	15.4	58.6	14.5	1.5	[−1.26	4.21]
Richest	362	41.3	61.0	13.8	3.9***	[1.89	5.90]
Occupation							
Agricultural labor	135	15.4	57.1	14.2	0.0	[0	0]
Casual labor	57	6.5	55.3	15.2	−1.9	[−6.17	2.46]
Salaried worker	92	10.5	61.5	12.4	4.4*	[0.71	8.10]
Self-employed in petty trade	166	18.9	61.3	13.5	4.2**	[1.05	7.39]
Self-employed small scale industry	25	2.9	61.5	13.0	4.4	[−1.55	10.4]
Unemployed/homemaker	391	44.6	58.4	14.2	1.2	[−1.49	3.97]
Other	11	1.3	58.4	12.2	1.3	[−7.32	9.81]
Partner’s occupation							
Agricultural labor	173	19.7	58.9	14.3	0.0	[0	0]
Casual labor	152	17.4	56.8	12.4	−2.2	[−5.19	0.81]
Salaried worker	148	16.9	61.9	13.7	3.0	[−0.057	5.98]
Self-employed in petty trade	119	13.6	63.7	12.8	4.8**	[1.55	7.98]
Self-employed small scale industry	74	8.4	55.4	14.2	−3.6	[−7.30	0.20]
Unemployed/homemaker	20	2.3	64.8	12.4	5.8	[−0.56	12.2]
Other	3	0.3	52.7	20.1	−6.3	[−22.0	9.43]
No partner	187	21.3	56.3	14.6	−2.6	[−5.47	0.22]
Partner’s education							
No school/Primary	308	35.9	58.9	13.8	0.0	[0	0]
Post-primary/Vocational/Secondary	225	26.2	59.1	13.8	0.1	[−2.24	2.53]
College or above	139	16.2	62.8	12.9	3.9**	[1.08	6.63]
No partner	187	21.8	56.3	14.6	−2.6*	[−5.11	−0.074]
Health Insurance							
No	722	82.3	57.8	13.9	0.0	[0	0]
Yes	155	17.7	64.1	13.3	6.3***	[3.87	8.67]
Participation in household decisions							
Low participation	440	50.2	58.3	14.7	0.0	[0	0]
High participation	437	49.8	59.6	13.2	1.3	[−0.61	3.10]
Attitude towards domestic violence							
DM tolerant	395	45.0	58.4	13.1	0.0	[0	0]
DM Intolerant	482	55.0	59.4	14.7	1.0	[−0.86	2.87]
Experience domestic violence							
No	429	48.9	61.5	14.3	0.0	[0	0]
Yes	448	51.1	56.5	13.2	−5.0***	[−6.84	−3.18]
Delivery facility type							
Public hospital	404	46.1	57.2	13.9	0.0	[0	0]
Health Center	362	41.3	59.3	13.7	2.1*	[0.13	4.07]
Mission/Private facility	111	12.7	63.9	14.1	6.7***	[3.80	9.62]
Delivery provider							
Nurse/Midwife	656	74.8	58.7	14.0	0.0	[0	0]
Doctor	83	9.5	61.9	14.7	3.2*	[0.051	6.43]
Clinical officer	54	6.2	54.9	11.6	−3.9	[−7.72	0.031]
Non-skilled attendant	21	2.4	62.4	12.9	3.7	[−2.39	9.75]
1plus skilled providers	63	7.2	60.0	14.2	1.3	[−2.28	4.95]
Delivery provider sex							
Male	329	37.5	59.1	13.5	0.0	[0	0]
Female	514	58.6	58.4	14.1	−0.7	[−2.61	1.26]
Both	34	3.9	66.1	14.8	7.0**	[2.08	11.9]
Pregnancy complications							
No	494	56.3	58.2	14.0	0.0	[0	0]
Yes	383	43.7	60.0	13.9	1.8	[−0.087	3.65]
Severe pregnancy complication							
No	616	70.2	58.3	13.9	0.0	[0	0]
Yes	261	29.8	60.5	14.1	2.3*	[0.22	4.28]
Past facility delivery							
No	342	39.0	58.1	14.6	0.0	[0	0]
Yes	535	61.0	59.5	13.6	1.5	[−0.46	3.35]
Trimester of first ANC							
First trimester	261	29.8	59.1	13.8	0.0	[0	0]
Second trimester	536	61.3	59.0	14.2	−0.2	[−2.23	1.92]
Third trimester	72	8.2	58.5	13.4	−0.6	[−4.28	3.04]
No ANC	6	0.7	51.3	15.6	−7.8	[−19.1	3.56]
Number of ANC visits							
No ANC	6	0.7	51.3	15.6	−6.9	[− 18.2	4.41]
Less than 4	281	32.2	58.2	14.0	0.0	[0	0]
4 or 5	485	55.6	59.4	14.1	1.2	[−0.85	3.26]
6 plus	100	11.5	59.3	13.3	1.1	[−2.10	4.29]
Tribe							
Luo	584	66.6	58.0	12.8	0.0	[0	0]
Kuria	208	23.7	61.8	16.3	3.8***	[1.63	6.04]
Other	85	9.7	58.6	15.1	0.6	[−2.61	3.74]
Religion							
Catholic	242	27.6	58.6	14.2	0.0	[0	0]
Protestant/Pentecostal	191	21.8	59.1	14.8	0.5	[−2.17	3.16]
Seventh Day Adventist	263	30.0	59.6	14.5	1.0	[−1.45	3.45]
Other Christian	166	18.9	58.6	11.8	0.0	[−2.77	2.77]
Muslim/other religion	15	1.7	55.1	15.1	−3.5	[−10.8	3.85]
Postpartum length							
Less than 1 week	75	8.6	66.1	15.1	0.0	[0	0]
1 week or more	802	91.4	58.3	13.7	−7.8***	[− 11.1	−4.50]
Postpartum length							
less than 5 weeks	426	48.6	60.1	14.7	0.0	[0	0]
5 weeks or more	451	51.4	57.9	13.2	−2.1*	[−4.00	− 0.29]
Place of Interview							
Health facility	356	40.6	60.7	14.2	0.0	[0	0]
In the community/a home	521	59.4	57.7	13.8	−3.0**	[−4.86	−1.10]

CI = 95% confidence intervals: * *p* < 0.05; ** *p* < 0.01; *** *p* < 0.001

### Bivariate

Selected bivariate statistics are shown in Table 1 to the right of the univariate statistics. Significant differences exist in PCMC scores by socioedemographic and facility characteristics. Not accounting for other factors women who are married, college educated, literate, wealthier, hold salaried jobs or have a trade, and have health insurance reported, on average, higher PCMC than women who are unmarried, less educated, illiterate, poorer, working in agriculture, and have no health insurance respectively. Mean PCMC score for unemployed women is 57 compared to 63 for those employed and that for women in the lowest wealth quintile is about 56 compared to 60 for those in the highest quintile. Women whose partners are college educated and have a trade reported higher PCMC than those whose partners have less education and are farmers respectively. Women who have experienced domestic violence and those who experienced severe pregnancy complications reported lower PCMC than those who did not experience these respectively. Additionally, women who delivered in health centers or private facilities, were assisted by doctors and by two providers of different sexes, reported higher PCMC than those who delivered in public hospitals, and were assisted by nurses, midwifes, or clinical officers and by only male or female providers, respectively. Kurias reported higher PCMC than Luos. Finally, women reported higher PCMC with increasing postpartum length and when interviewed in their communities compared to in health facilities.

### Multivariable

The full model shown in the last column of Table 2 shows that, net of other factors, women who are employed and those who can write very well scored about five points higher on the PCMC scale than those who are not employed and cannot write well, respectively. Also women in the highest wealth quintile scored about three points higher than those in the lowest quintiles. In addition, women who delivered in health centers and private facilities scored about four and six points higher respectively on the scale than those who delivered in public hospitals; and those who had both male and female providers at delivery scored about six points higher than those who had only male or female providers. The effects of marital status, tribe, and timing and location of the interview also persist after controlling for other factors.

**Table 2:** Multivariable Linear Regression of PCMC score on selected predictors, PQCC data 2016

	Nested models								
	PM1: All except employment			PM3: All except facility and provider characteristics			Full Model		
	*Coeff.*	*CI*		*Coeff.*	*CI*		*Coeff.*	*CI*	
Household wealth									
Poorest/poorer	0	[0	0]	0	[0	0]	0	[0	0]
Middle	1.56	[−1.04	4.17]	0.91	[−1.71	3.53]	1.42	[−1.16	4.00]
Richer/richest	3.99***	[1.88	6.11]	2.04	[−0.085	4.17]	2.90**	[0.76	5.05]
Employed				5.19***	[3.09	7.29]	4.69***	[2.63	6.76]
Literacy									
Cannot read	0	[0	0]	0	[0	0]	0	[0	0]
Yes, with some difficulty	4.67	[−0.47	9.81]	5.11	[−0.072	10.3]	5.17*	[0.083	10.3]
Yes, very well	4.64	[−0.18	9.46]	5.54*	[0.66	10.4]	5.52*	[0.73	10.3]
Experienced domestic violence	−5.24***	[−7.07	−3.41]	−4.68***	[−6.52	−2.84]	−4.97***	[−6.78	−3.16]
Facility type									
Public Hospital	0	[0	0]				0	[0	0]
Health center	4.62***	[2.66	6.58]				4.46***	[2.52	6.40]
Mission/Private	6.31***	[3.52	9.10]				5.74***	[2.97	8.51]
Provider sex									
Male	0	[0	0]				0	[0	0]
Female	−0.35	[−2.18	1.49]				−0.27	[−2.08	1.54]
Both	6.50**	[1.84	11.2]				6.43**	[1.82	11.0]
Currently married	3.69***	[1.55	5.83]	3.44**	[1.27	5.60]	3.20**	[1.07	5.33]
Tribe									
Luo	0	[0	0]	0	[0	0]	0	[0	0]
Kuria	5.19***	[2.98	7.39]	4.10***	[1.92	6.29]	5.06***	[2.87	7.24]
Other	−0.22	[−3.23	2.79]	0.19	[−2.84	3.21]	−0.26	[−3.23	2.72]
Interviews in community	−2.84**	[−4.67	−1.00]	− 2.30*	[−4.13	−0.48]	−2.90**	[−4.72	−1.09]
Postpartum length =/> 1 week	−5.24**	[−8.45	−2.03]	−5.53***	[−8.76	− 2.30]	− 5.09**	[−8.27	−1.91]
Constant	54.9***	[48.8	61.0]	56.5***	[50.6	62.5]	53.7***	[47.6	59.8]
N	877			877			877		
R-squared	0.14			0.13			0.16		

CI = 95% confidence intervals: * *p* < 0.05; ** *p* < 0.01; *** *p* < 0.001

### Mediation results

The results of the mediation analysis are also shown in Table 2. The first two models are the partial models (PMs), which are the models with all predictors except the potential mediating variables. PM1 includes all predictors except employment status, and the coefficient for wealth here is its total effect on PCMC. PM2 includes all predictors except the facility and provider characteristics, and the coefficient for wealth and employment status are their total effects on PCMC. The differences between the coefficients in these partial models and the full model are the mediated effects. Examining PM1 and the full model shows that, when a woman’s employment is not accounted for, women in wealthier households score about four PCMC points higher than those in poorer households. This decreases by about one point when employment is added to the model, suggesting about 25% [(3.99–2.99)/3.99*100] of the difference in PCMC between the richest and poorest women is accounted by their employment status. When the reverse is examined, about 21% of the effect of working is accounted for by wealth [(5.31–4.69)/5.31*100]. Examining PM2 and the full model shows that only about 10% [(5.19–4.69)/5.19*100 = 9.6%] of the employment effect is accounted by the facility and provider characteristics. Notably, wealth is significant in the full model but not in PM2, suggesting the facility and provider characteristics do not mediate the wealth effect, but there might be some suppression or moderation going on—shown in the moderation analysis.

### Moderation results

**Fig. 1:**
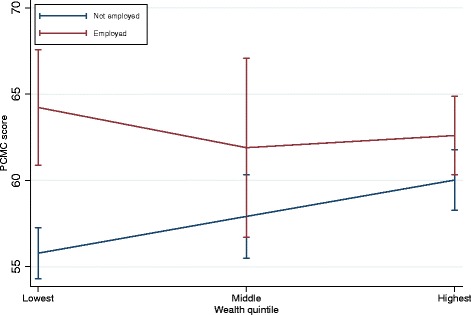
PCMC score by wealth and employment status

The first conditional model in Table 3 shows the interaction between women’s employment and household wealth. The coefficients for wealth in this model are the effects of wealth among women who are not employed (the reference group). This shows that among unemployed women, those in households in the highest wealth quintiles have a PCMC score of about 4 points higher than those in the lowest wealth quintiles. But the difference between those in the middle and lowest quintiles is not significant. The coefficient for employment shows that among the poorest women, those who are employed have a PCMC score about eight points higher than those not employed. The coefficients for the interaction terms are the differences in the slopes for employment and PCMC by wealth. They show that the magnitude of the effect of working on PCMC among the poorest women differs from the richest women, but not from those in the middle wealth quintile. The plot of the interaction in Fig. 1 illustrates these results. It shows that PCMC scores decrease between the poorest and middle wealth quintiles among employed women but increase among the unemployed. Both however increase between the middle and richest quintiles. The confidence intervals for the wealth quintiles among employed women, however, overlap, implying the downward trend is not significant. But there is a significant difference in PCMC scores by employment status among the poorest women, which does not exist at higher levels of wealth. To summarize, the results from this conditional model show that unemployed women from poor households receive the lowest quality PCMC.

**Table 3:** Multivariable linear Regression of PCMC score on selected predictors, PQCC data 2016

	Conditional models					
	Wealth/Employment Interaction			Wealth/Facility Interaction		
	*Coeff.*	*CI*		*Coeff.*	*CI*	
Employed	8.44***	[4.86	12.0]	4.80***	[2.73	6.86]
Household wealth						
Poorest/poorer	0	[0	0]	0	[0	0]
Middle	2.13	[−0.69	4.96]	3.79	[−0.048	7.63]
Richer/richest	4.24***	[1.85	6.63]	5.71***	[2.74	8.69]
Facility type						
Public hospital	0	[0	0]	0	[0	0]
Health center	4.46***	[2.52	6.39]	7.69***	[4.87	10.5]
Mission/Private	5.96***	[3.19	8.74]	5.49*	[0.54	10.4]
Emp*Wealth						
Employed*Poorest/poorer	0	[0	0]			
Employed*Middle	−4.46	[−11.2	2.31]			
Employed*Richer/richest	−5.86*	[−10.4	−1.32]			
Facility*Wealth						
Health center*Poorest/poorer				0	[0	0]
Health center*Middle				−5.97*	[−11.3	−0.61]
Health center*Richer/richest				−6.00**	[−10.2	−1.81]
Mission/Private*Poorest/poorer				0	[0	0]
Mission/Private*Middle				6.21	[−2.94	15.4]
Mission/Private*Richer/richest				−1.12	[−7.26	5.02]
Constant	52.6***	[46.4	58.7]	51.0***	[44.7	57.4]
N	877			877		
R-squared	0.17			0.18		

CI = 95% confidence intervals: * *p* < 0.05; ** *p* < 0.01; *** *p* < 0.001. All models include literacy, domestic violence experience, provider sex, marital status, tribe, post-partum length and place of interview

**Fig. 2:**
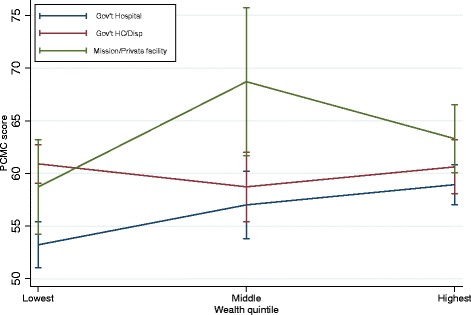
PCMC score by wealth and facility type

The second conditional model in Table 3 shows the interaction between household wealth and facility type. Here we see that the wealth differences exist even within the same types of facilities. In public hospitals, women in households in the highest wealth quintiles score about six points higher on PCMC than those in the lowest quintiles. Also among the poorest women, those who delivered in health centers and in private facilities have a PCMC score of about eight and five points higher respectively than those who delivered in public hospitals. The coefficients for the interaction terms show that the magnitude of the effect of facility type on PCMC differs by wealth, as illustrated in Fig. 2. Figure 2 shows that PCMC scores decrease between the poorest and middle wealth quintiles in health centers but increases in public hospitals and private facilities. PCMC scores in both health centers and public hospitals however increase between the middle and richest quintiles, with that of private facilities decreasing. The confidence intervals for the wealth quintiles are however overlapping for health centers and private facilities, implying the effect of wealth is not significant within health centers and private facilities. But the effect of wealth is significant in the public hospitals, especially between the lowest and highest wealth quintile. There is also a significant difference in PCMC scores between health centers and hospitals among the poorest women. The difference in PCMC scores between private facilities and public hospitals are not significantly different among the poorest women. But it is significant for women in the middle wealth quintile. At the highest wealth quintile, although the PCMC scores for health centers, and in particular private facilities are still higher than that for hospitals these differences are not statistically significant. In summary, poor women who deliver in higher-level facilities receive the lowest quality PCMC.

## Discussion

To our knowledge this is the first study to quantitatively examine factors associated with PCMC in SSA. We find that women from wealthier households, and those who are employed receive higher PCMC than those from poorer households and those who are not employed. But the effect of employment is strongest for the poorest women, with unemployed women from poor households receiving the lowest quality care. Also, women who delivered in health centers and private facilities and those who were attended to by two or more providers of different sexes received higher PCMC than those who delivered in public hospitals and who were attended to by only male or female providers. The difference in PCMC between health centers and hospitals is however only significant among the poorest women and that between private facilities and hospitals is only significant among women in the middle wealth quintiles. There is no difference between private facilities and health centers. Other factors that significantly predict PCMC, net of other factors, are marital status, literacy, experience of domestic violence, and tribe. Finally, we find that women who were interviewed within a few weeks of birth and those who were interviewed in health facilities reported lower PCMC than those who were interviewed several weeks after births in their homes.

These findings are consistent with prior studies on quality of maternal health services, but differ in some ways. First, similar to studies on quality of antenatal care [34, 35, 63], we find disparities in quality of care by SES as measured by household wealth. Education also predicted PCMC in the bivariate model, but did not make it into the final multivariable models because its effect was not significant and it did not improve the model: likely due to its association with other measures in the model like wealth, employment, and literacy. Through mediation and moderation analyses, our study goes further than the few quantitative studies examining predictors of quality of maternal health care to show that even among women of similar SES, gradients of care exist. Among the poorest women, their employment status and where they seek care greatly affects their care. Given the much smaller proportions of poor women who use health facilities, this study extends evidence that poor experience reinforces aversion to using these facilities [11, 13, 39].

Additionally, we find women’s empowerment to be important for quality of care. While many studies have examined empowerment and use of maternal health services, none to our knowledge have examined empowerment and quality of maternal health services in SSA, although many theorize empowerment affects quality of care [45]. This study extends the literature on how women’s empowerment can affect health outcomes. Of note is the interaction between women’s economic empowerment and household wealth discussed above. The conditional effect is potentially due to women from wealthier households being accorded a certain level of respect by health providers irrespective of their personal standing and ability to advocate for themselves. Women from wealthier households may also have other people within their households advocating for respectful care for them. On the other hand, while women from poorer households are more likely to be mistreated, being employed increases women’s economic empowerment, which enables them to access care where they will be treated better [45, 52]. Employment could also be a proxy for other forms of empowerment including sociocultural, cognitive, and psychosocial empowerment, because employment does expand women’s “ability to make strategic life choices in a context where this ability was previously denied to them” [46, 53, 55]. In this case we hypothesize we are seeing an effect of women’s ability to advocate for respectful and responsive care for themselves. The association between domestic violence and PCMC also provides support for the theorized relationship between gender based violence and mistreatment in health facilities [56]. The finding suggests women who experienced violence at home may be doubly traumatized by mistreatment in health facilities.

Our finding that PCMC is higher in lower-level and private facilities is also consistent with studies that find higher interpersonal quality of care and satisfaction with services in private than public facilities [26, 37, 40]. Studies on clinical quality measures however find higher quality of care in hospitals than in health centers [26, 34]. Higher PCMC scores in lower level facilities is potentially due to closer ties with women, since they serve smaller communities where providers may know women presenting to their facilities. Private facilities may also pay greater attention to client satisfaction to attract more patients. In addition, both health centers and private facilities usually have lower patient volumes which decreases the strain on their interactions with patients. That wealth only makes a difference in public hospitals is therefore likely because PCMC is generally poor in these higher-level facilities, but wealthier families are accorded better care because of their status. Similarly, that the difference between health centers and public hospitals is only significant among the poorest women is likely because the closer ties in health centers that improves PCMC, even for the poorest women, is absent at the higher level facilities, which are often outside of the immediate communities where the poorest women live. Put differently, the findings suggest exceptionalism in PCMC is usually positive, with some groups receiving better care than the norm, rather than negative, where the norm is good care and one group is singled out for worse treatment.

The effect of marital status is expected, as qualitative studies suggest adolescent or unmarried women experience mistreatment more frequently, as pregnancy is often viewed as appropriate only in the context of marriage [21]. The effect of age on the other hand was not statistically significant even though the mean PCMC score was lower for women less than 20 years compared to those 20 years and older. The small number of adolescents in our sample may have contributed to the non-significant effect of age. Also age is associated with marital status and the measures of SES. It is unclear why Luos report lower PCMC than Kurias. A potential reason is discrimination by tribe within the health facilities. Also Kuria women were more likely to be accompanied to the facility than Luo women, and having a labor companion has been found to be associated with less mistreatment [64]. Finally the difference by post-partum length and place of interview are expected. Women are less likely to report negative experiences when interviewed immediately following delivery, compared to when interviewed five to ten weeks postpartum as the joy of having just delivered a healthy baby often overshadows their negative experience. Time allows women to process their experiences independent of the pregnancy outcome [65]. Women are also more comfortable talking about negative experiences at the health facilities in their own home as opposed to in the facility.

The study has potential limitations. First the measure of PCMC is based on self-report. Social desirability and recall bias are thus potential limitations. Second, we operationalized SES and empowerment using measures such as household wealth, woman’s employment, and literacy characteristics. We may however be missing other relevant factors that characterize a woman’s SES and empowerment in the setting. Third, small sample sizes for groups such as adolescents, and people working in or with relations working in facilities, may have contributed non-significant effects of some factors thought to contribute to differential care. Similarly, small sample sizes resulted in large confidence intervals for some predictors such as delivery in a private facility in the conditional models and potentially unstable estimates for some, such as literacy. We excluded 17 observations (1.9%) missing on one or more key variables. Women excluded were not significantly different from those included except on parity and experience of domestic violence (women excluded were more likely to be primiparous and less likely to have experienced domestic violence). Furthermore, we are unable to adequately account for other characteristics of facilities and providers that may affect the care they provide. Finally, the quantitative data do not offer the qualitative nuances useful for understanding the pathways through which the various predictors affect quality of care that would help shape interventions.

Despite these limitations, this study makes valuable contributions to existing research by using a person-centered approach and a validated tool to understand disparities in quality of care. Our study is one of the few studies to empirically assess disparities in PCMC. These findings highlight inequalities common in person-centered care during delivery in Kenya. It is known that poorer households delay or avoid seeking care from the formal sector. In almost all countries, poor women are less likely to seek skilled care at birth [10, 11, 39]. In Kenya, children of poor families are 25% more likely to fall ill with respiratory infections, but 17% less likely to seek care once ill [12]. The “three delays”—the decision to seek, reach, and receive care—are among the primary drivers of maternal and neonatal mortality [41]. A study in Uganda found that delays in seeking care were responsible for half of all newborn deaths [66]. Past experiences of poor treatment and poor treatment received by family, friends, and neighbors, along with the accumulated effects of social stigmatization and disenfranchisement all contribute to these delays. The unequal distribution of delays is likely influenced by differential experiences, which in some settings is a more important driver of care-seeking than clinical capacity within the facility [67]. This makes PCMC both a human right and a critical input for maternal and neonatal outcomes.

## Conclusions

Our study has shown, with a validated and sensitive scale, that sub-populations in Kenya receive unequal care. This work adds accuracy and refinement to the findings from past studies. Women who are poor, unemployed, illiterate, and unmarried suffer greater indignities and receive less information about their care than women who are better off and more empowered. Our results provide definitive data to show that specific groups, including poor, unemployed, illiterate, younger, and unmarried women, need special attention simply to rise to the equivalent level of treatment given to other women. Our work has also raised a question about where the ‘best’ care for women might take place during delivery. It is often assumed that providing the best clinical care will assure the best outcomes. Our study suggests that for low-risk births, the better clinical care available in larger hospitals, which have the highest levels of staffing and clinical infrastructure, must be measured against the better treatment to be had at health centers or private hospitals. Targeted PCMC interventions in these higher-level facilities are needed. There is growing appreciation that quality of care is limiting rapid reduction in maternal and newborn deaths. This study underscores that person-centered care is lowest for those most at risk, and so must be considered and addressed concomitantly with more traditional clinical and infrastructure-related dimensions of care. Attention to disparities in person-centerd care will also be critical to attaining health equity and the SDG commitment that “no one is left behind”.

## Additional files

Additional file 1: Distribution of person-centered maternity care variables. Table showing distribution of individual items used to create the PCMC scores. (DOCX 41 kb)

Additional file 2: Empowerment questions. Shows questions used to create empowerment scores. (PDF 151 kb)

## Abbreviations

DHS: Demographic and Health Survey
OLS: Ordinary least squares
PCMC: Person-centered maternity care
PM: Partial models
SES: Socioeconomic status
SSA: Sub-Saharan Africa
