# Supplementary material for: Predictors of person-centered maternity care: the role of socioeconomic status, empowerment, and facility type
Source: BMC Health Serv Res. 2018 May 11;18:360. doi: 10.1186/s12913-018-3183-x (PMC5948900; doi:10.1186/s12913-018-3183-x)
Supplement: Supplementary file 1 — Distribution of person-centered maternity care variables. Table showing distribution of individual items used to create the PCMC scores. (DOCX 41 kb) [file 12913_2018_3183_MOESM1_ESM.docx]

| **Additional file 1: Distribution of person-centered maternity care variables** | | | |
| --- | --- | --- | --- |
|  | No. | % |  |
| How did you feel about the amount of time you waited? Would you say it was … |  |  |  |
| 0 Very short | 525 | 61.3 |  |
| 1 Somewhat short | 195 | 22.8 |  |
| 2 Somewhat long | 85 | 9.9 |  |
| 3 Very long | 52 | 6.1 |  |
|  |  |  |  |
| During your time in the health facility did the doctors, nurses, or other health care providers introduce themselves to you when they first came to see you? |  |  |  |
| 0 No, none of them | 658 | 76.8 |  |
| 1 Yes, a few of them | 107 | 12.5 |  |
| 2 Yes, most of them | 41 | 4.8 |  |
| 3 Yes, all of them | 51 | 6 |  |
|  |  |  |  |
| Did the doctors, nurses, or other health care providers call you by your name? |  |  |  |
| 0 No, never | 230 | 26.8 |  |
| 1 Yes, a few times | 165 | 19.3 |  |
| 2 Yes, most of the time | 137 | 16 |  |
| 3 Yes, all the time | 325 | 37.9 |  |
|  |  |  |  |
| Did the doctors, nurses, or other staff at the facility treat you with respect? |  |  |  |
| 0 No, never | 17 | 1.9 |  |
| 1 Yes, a few times | 76 | 8.9 |  |
| 2 Yes, most of the time | 250 | 29.2 |  |
| 3 Yes, all the time | 514 | 60 |  |
|  |  |  |  |
| Did the doctors, nurses, and other staff at the facility treat you in a friendly manner? |  |  |  |
| 0 No, never | 23 | 2.7 |  |
| 1 Yes, a few times | 94 | 10.9 |  |
| 2 Yes, most of the time | 238 | 27.8 |  |
| 3 Yes, all the time | 502 | 58.6 |  |
|  |  |  |  |
|  |  |  |  |
| Did you feel the doctors, nurses, or other health providers shouted at you, scolded, insulted, threatened, or talked to you rudely? |  |  |  |
| 0 No, never | 763 | 89 |  |
| 1 Yes, once | 56 | 6.5 |  |
| 2 Yes, a few times | 22 | 2.6 |  |
| 3 Yes, many times | 16 | 1.9 |  |
|  |  |  |  |
| Did you feel like you were treated roughly like pushed, beaten, slapped, pinched, physically restrained, or gagged? |  |  |  |
| 0 No, never | 819 | 95.6 |  |
| 1 Yes, once | 24 | 2.8 |  |
| 2 Yes, a few times | 9 | 1.1 |  |
| 3 Yes, many times | 5 | 0.6 |  |
|  |  |  |  |
|  |  |  |  |
|  |  |  |  |
| During examinations in the labor room, were you covered up with a cloth or blanket or screened with a curtain so that you did not feel exposed? |  |  |  |
| 0 No, never | 168 | 19.6 |  |
| 1 Yes, a few times | 57 | 6.7 |  |
| 2 Yes, most of the time | 112 | 13.1 |  |
| 3 Yes, all the time | 510 | 59.5 |  |
| 4 Not applicable | 10 | 1.2 |  |
|  |  |  |  |
| Do you feel like your health information was or will be kept confidential at this facility? |  |  |  |
| 0 No, never | 48 | 5.6 |  |
| 1 Yes, a few times | 105 | 12.3 |  |
| 2 Yes, most of the time | 263 | 30.7 |  |
| 3 Yes, all the time | 441 | 51.5 |  |
|  |  |  |  |
| Did you feel like the doctors, nurses or other staff at the facility involved you in decisions about your care? |  |  |  |
| 0 No, never | 160 | 18.7 |  |
| 1 Yes, a few times | 111 | 13 |  |
| 2 Yes, most of the time | 171 | 20 |  |
| 3 Yes, all the time | 344 | 40.1 |  |
| 4 Did not have to make any decisions | 71 | 8.3 |  |
|  |  |  |  |
| Did the doctors, nurses or other staff at the facility ask your permission/consent before doing procedures on you? |  |  |  |
| 0 No, never | 303 | 35.4 |  |
| 1 Yes, a few times | 118 | 13.8 |  |
| 2 Yes, most of the time | 195 | 22.8 |  |
| 3 Yes, all the time | 241 | 28.1 |  |
|  |  |  |  |
| During the delivery, do you feel like you were able to be in the position of your choice? |  |  |  |
| 0 No, never | 600 | 70 |  |
| 1 Yes, for a short time | 105 | 12.3 |  |
| 2 Yes, most of the time | 74 | 8.6 |  |
| 3 Yes, all the time | 78 | 9.1 |  |
|  |  |  |  |
| Did the doctors, nurses or other staff at the facility speak to you in a language you could understand? |  |  |  |
| 0 No, never | 21 | 2.5 |  |
| 1 Yes, a few times | 65 | 7.6 |  |
| 2 Yes, most of the time | 181 | 21.1 |  |
| 3 Yes, all the time | 590 | 68.8 |  |
|  |  |  |  |
| Did the doctors and nurses explain to you why they were doing examinations or procedures on you? |  |  |  |
| 0 No, never | 232 | 27.1 |  |
| 1 Yes, a few times | 119 | 13.9 |  |
| 2 Yes, most of the time | 215 | 25.1 |  |
| 3 Yes, all the time | 291 | 34 |  |
|  |  |  |  |
| Did the doctors and nurses explain to you why they were giving you any medicine? |  |  |  |
| 0 No, never | 141 | 16.5 |  |
| 1 Yes, a few times | 104 | 12.1 |  |
| 2 Yes, most of the time | 208 | 24.3 |  |
| 3 Yes, all the time | 332 | 38.7 |  |
| 4 Did not get any medicine | 72 | 8.4 |  |
|  |  |  |  |
| Did you feel you could ask the doctors, nurses or other staff at the facility any questions you had? |  |  |  |
| 0 No, never | 190 | 22.2 |  |
| 1 Yes, a few times | 205 | 23.9 |  |
| 2 Yes, most of the time | 184 | 21.5 |  |
| 3 Yes, all the time | 278 | 32.4 |  |
|  |  |  |  |
| Did the doctors and nurses at the facility talk to you about how you were feeling? |  |  |  |
| 0 No, never | 113 | 13.2 |  |
| 1 Yes, a few times | 257 | 30 |  |
| 2 Yes, most of the time | 220 | 25.7 |  |
| 3 Yes, all the time | 267 | 31.2 |  |
|  |  |  |  |
| Did the doctors, nurses or other staff at the facility try to understand your anxieties? |  |  |  |
| 0 No, never | 199 | 23.2 |  |
| 1 Yes, a few times | 199 | 23.2 |  |
| 2 Yes, most of the time | 146 | 17 |  |
| 3 Yes, all the time | 180 | 21 |  |
| 4 I did not have any anxieties or fears | 133 | 15.5 |  |
| Total | 857 | 100 |  |
|  |  |  |  |
| When you needed help, did you feel the doctors, nurses or other staff at the facility paid attention? |  |  |  |
| 0 No, never | 38 | 4.4 |  |
| 1 Yes, a few times | 120 | 14 |  |
| 2 Yes, most of the time | 324 | 37.8 |  |
| 3 Yes, all the time | 375 | 43.8 |  |
|  |  |  |  |
|  |  |  |  |
|  |  |  |  |
| Do you feel the doctors or nurses did everything they could to help control your pain? |  |  |  |
| 0 No, never | 324 | 37.8 |  |
| 1 Yes, a few times | 133 | 15.5 |  |
| 2 Yes, most of the time | 187 | 21.8 |  |
| 3 Yes, all the time | 213 | 24.9 |  |
|  |  |  |  |
|  |  |  |  |
| Were you allowed to have someone you wanted (outside of staff at the facility, such as family or friends) to stay with you during labor? |  |  |  |
| 0 No, never | 162 | 18.9 |  |
| 1 Yes, a few times | 103 | 12 |  |
| 2 Yes, most of the time | 230 | 26.8 |  |
| 3 Yes, all the time | 357 | 41.7 |  |
| 4 I did not want someone to stay with me | 5 | 0.6 |  |
|  |  |  |  |
| Were you allowed to have someone you wanted to stay with you during delivery? |  |  |  |
| 0 No, never | 524 | 61.1 |  |
| 1 Yes, a few times | 72 | 8.4 |  |
| 2 Yes, most of the time | 110 | 12.8 |  |
| 3 Yes, all the time | 138 | 16.1 |  |
| 4 I did not want someone to stay with me | 13 | 1.5 |  |
|  |  |  |  |
| Do you think there was enough health staff in the facility to care for you? |  |  |  |
| 0 No, never | 109 | 12.7 |  |
| 1 Yes, a few times | 119 | 13.9 |  |
| 2 Yes, most of the time | 250 | 29.2 |  |
| 3 Yes, all the time | 379 | 44.2 |  |
|  |  |  |  |
| Did you feel the doctors, nurses or other staff at the facility took the best care of you? |  |  |  |
| 0 No, never | 18 | 2.1 |  |
| 1 Yes, a few times | 77 | 9 |  |
| 2 Yes, most of the time | 307 | 35.8 |  |
| 3 Yes, all the time | 455 | 53.1 |  |
|  |  |  |  |
| Did you feel you could completely trust the doctors, nurses or other staff at the facility with regards to your care? |  |  |  |
| 0 No, never | 24 | 2.8 |  |
| 1 Yes, a few times | 84 | 9.8 |  |
| 2 Yes, most of the time | 293 | 34.2 |  |
| 3 Yes, all the time | 456 | 53.2 |  |
|  |  |  |  |
|  |  |  |  |
|  |  |  |  |
| Thinking about the labor and postnatal wards, did you feel the health facility was croweded? |  |  |  |
| 0 No, never | 400 | 46.7 |  |
| 1 Yes, a few times | 177 | 20.7 |  |
| 2 Yes, most of the time | 140 | 16.3 |  |
| 3 Yes, all the time | 140 | 16.3 |  |
|  |  |  |  |
| Thinking about the wards, washrooms and the general environment of the health facility, will you say the facility was very clean, clean, dirty, or very dirty? |  |  |  |
| 0 Very dirty | 8 | 0.9 |  |
| 1 Dirty | 99 | 11.6 |  |
| 2 Clean | 604 | 70.5 |  |
| 3 Very clean | 146 | 17 |  |
|  |  |  |  |
| Was there water in the facility? |  |  |  |
| 0 No, never | 33 | 3.9 |  |
| 1 Yes, a few times | 65 | 7.6 |  |
| 2 Yes, most of the time | 224 | 26.1 |  |
| 3 Yes, all the time | 535 | 62.4 |  |
|  |  |  |  |
| Was there electricity in the facility? |  |  |  |
| 0 No, never | 43 | 5 |  |
| 1 Yes, a few times | 70 | 8.2 |  |
| 2 Yes, most of the time | 254 | 29.6 |  |
| 3 Yes, all the time | 490 | 57.2 |  |
|  |  |  |  |
| In general, did you feel safe in the health facility? |  |  |  |
| 1 Yes, a few times | 57 | 6.7 |  |
| 2 Yes, most of the time | 198 | 23.1 |  |
| 3 Yes, all the time | 602 | 70.2 |  |
|  |  |  |  |
| Total | 857 | 100 |  |
