# Supplementary material for: Predictors of person-centered maternity care: the role of socioeconomic status, empowerment, and facility type
Source: BMC Health Serv Res. 2018 May 11;18:360. doi: 10.1186/s12913-018-3183-x (PMC5948900; doi:10.1186/s12913-018-3183-x)
Supplement: Supplementary file 2 — Empowerment questions. Shows questions used to create empowerment scores. (PDF 151 kb) [file 12913_2018_3183_MOESM2_ESM.pdf]

Empowerment questions

Question	Response options	comment
EMPOWERMENT<br><br>Before we end, I would like to ask you questions about some other important aspects of a woman's life. I know that some of these questions are very personal. However, your answers are crucial for helping to understand the condition of women in Kenya. Let me assure you that your answers are completely confidential and will not be told to anyone and no one else will know that you were asked these questions.<br><br>Household decision making:<br>First I am going to ask you some questions about how decisions are made in your household		
Who usually decides how the money you earn will be used: mainly you, mainly your husband/partner, or<br>you and your husband/partner jointly?	1, 1 Respondent | 2, 2 Husband/Partner | 3, 3 Respondent And Husband/Partner Jointly | 6, 6 Other	(Use partner if respondent is not married)
Would you say that the money that you earn is more than what your husband/partner earns, less than what he earns, or about the same?	1, 1 More Than Him | 2, 2 Less Than Him | 3, 3 About The Same | 4, 4 Husband/Partner Doesn't Bring In Any Money | 8, 8 Don'tknow	
Who usually decides how your husband's/partner's earnings will be used:<br>you, your husband/partner, or you and your husband/partner jointly?	1, 1 Respondent | 2, 2 Husband/Partner | 3, 3 Respondent And Husband/Partner Jointly | 4, 4 Husband/Partner Has No Earnings | 6, 6 Other	
Who usually makes decisions about health care for yourself:<br>you, your husband/partner, you and your husband/partner jointly, or someone else?	1, 1 Respondent | 2, 2 Husband/Partner | 3, 3 Respondent And Husband/Partner Jointly | 4, 4 Someone Else	
Who usually makes decisions about making major household purchases?	1, 1 Respondent | 2, 2 Husband/Partner | 3, 3 Respondent And Husband/Partner Jointly | 4, 4 Someone Else	

Who usually makes decisions about making purchases for daily household needs?	1, 1 Respondent | 2, 2 Husband/Partner | 3, 3 Respondent And Husband/Partner Jointly | 4, 4 Someone Else	
Who usually makes decisions about visits to your family or relatives?	1, 1 Respondent | 2, 2 Husband/Partner | 3, 3 Respondent And Husband/Partner Jointly | 4, 4 Someone Else	
Who usually makes decisions about what food should be cooked each day?	1, 1 Respondent | 2, 2 Husband/Partner | 3, 3 Respondent And Husband/Partner Jointly | 4, 4 Someone Else	
DO NOT ASK ALOUD.<br>PRESENCE OF OTHERS AT THIS POINT<br>(PRESENT AND LISTENING, PRESENT BUT NOT LISTENING, OR NOT PRESENT)	0, 0 No one Present | 1, 1 Some one Present And Listening | 2, 2 Some one Present But Not Listening	
OBSERVE WHO IS PRESENT: DO NOT ASK ALOUD.	1, 1 Children <10 | 2, 2 Husband | 3, 3 Other males | 4, 4 Other females |	
Sometimes a husband is annoyed or angered by things that his wife does.<br>In your opinion, is a husband justified in hitting or beating his wife in the following situations:<br><br>If she goes out without telling him?	0, 0 No | 1, 1 Yes | 8, 8 Don't know	
If she neglects the children?	0, 0 No | 1, 1 Yes | 8, 8 Don't know	
If she argues with him?	0, 0 No | 1, 1 Yes | 8, 8 Don't know	
If she refuses to have sex with him?	0, 0 No | 1, 1 Yes | 8, 8 Don't know	
If she burns the food?	0, 0 No | 1, 1 Yes | 8, 8 Don't know	

DOMESTIC VIOLENCE<br><br>I am going to to ask some more questions about your relationship with your (last) husband/partner. If we should come to any question that you do not want to answer, just let me know and we will go on to the next question. I know that some of these questions are very personal. However, your answers are crucial for helping to understand the condition of women in Kenya. Let me assure you that your answers are completely confidential and will not be told to anyone and no one else will know that you were asked these questions. These are the last set of questions, so we will be done soon		
Does (did) your (last) husband/partner ever:<br><br>a) say or do something to humiliate you in front of others?	0, 0 No | 1, 1 Yes | 8, 8 Don't know | 9, 9 Refused	(Use partner if respondent is not married)
How often did this happen during the last 12 months: often, only sometimes, or not at all?	0, 0 Not at all | 1, 1 Only sometimes | 2, 2 Often | 8, 8 Don't know | 9, 9 Refused	
Does/Did he ever<br><br>b) threaten to hurt or harm you or someone close to you?	0, 0 No | 1, 1 Yes | 8, 8 Don't know | 9, 9 Refused	
How often did this happen during the last 12 months: often, only sometimes, or not at all?	0, 0 Not at all | 1, 1 Only sometimes | 2, 2 Often | 8, 8 Don't know | 9, 9 Refused	
Does/Did he ever<br><br>c) insult you or make you feel bad about yourself?	0, 0 No | 1, 1 Yes | 8, 8 Don't know | 9, 9 Refused	

How often did this happen during the last 12 months: often, only sometimes, or not at all?	0, 0 Not at all | 1, 1 Only sometimes | 2, 2 Often | 8, 8 Don't know | 9, 9 Refused	
(Does/did) your (last) husband/partner ever do any of the following things to you:<br><br>(a) Push you, shake you, or throw something at you?	0, 0 No | 1, 1 Yes | 8, 8 Don't know | 9, 9 Refused	
How often did this happen during the last 12 months: often, only sometimes, or not at all?	0, 0 Not at all | 1, 1 Only sometimes | 2, 2 Often | 8, 8 Don't know | 9, 9 Refused	
Does/Did he ever<br><br>(b) Slap you?	0, 0 No | 1, 1 Yes | 8, 8 Don't know | 9, 9 Refused	
How often did this happen during the last 12 months: often, only sometimes, or not at all?	0, 0 Not at all | 1, 1 Only sometimes | 2, 2 Often | 8, 8 Don't know | 9, 9 Refused	
Does/Did he ever<br><br>(c) Twist your arm or pull your hair?	0, 0 No | 1, 1 Yes | 8, 8 Don't know | 9, 9 Refused	
How often did this happen during the last 12 months: often, only sometimes, or not at all?	0, 0 Not at all | 1, 1 Only sometimes | 2, 2 Often | 8, 8 Don't know | 9, 9 Refused	
Does/Did he ever<br><br>(d) Punch you with his fist or with something that could hurt you?	0, 0 No | 1, 1 Yes | 8, 8 Don't know | 9, 9 Refused	
How often did this happen during the last 12 months: often, only sometimes, or not at all?	0, 0 Not at all | 1, 1 Only sometimes | 2, 2 Often | 8, 8 Don't know | 9, 9 Refused	

Does/Did he ever<br><br>(e) Kick you or drag you or beat you up?	0, 0 No | 1, 1 Yes | 8, 8 Don't know | 9, 9 Refused	
How often did this happen during the last 12 months: often, only sometimes, or not at all?	0, 0 Not at all | 1, 1 Only sometimes | 2, 2 Often | 8, 8 Don't know | 9, 9 Refused	
(f) Try to choke you or burn you on purpose?	0, 0 No | 1, 1 Yes | 8, 8 Don't know | 9, 9 Refused	
How often did this happen during the last 12 months: often, only sometimes, or not at all?	0, 0 Not at all | 1, 1 Only sometimes | 2, 2 Often | 8, 8 Don't know | 9, 9 Refused	
Does/Did he ever<br><br>(g) Threaten or attack you with a knife, gun, or any other weapon?	0, 0 No | 1, 1 Yes | 8, 8 Don't know | 9, 9 Refused	
How often did this happen during the last 12 months: often, only sometimes, or not at all?	0, 0 Not at all | 1, 1 Only sometimes | 2, 2 Often | 8, 8 Don't know | 9, 9 Refused	
Does/Did he ever<br><br>(h) Physically force you to have sexual intercourse even when you did not want to?	0, 0 No | 1, 1 Yes | 8, 8 Don't know | 9, 9 Refused	
How often did this happen during the last 12 months: often, only sometimes, or not at all?	0, 0 Not at all | 1, 1 Only sometimes | 2, 2 Often | 8, 8 Don't know | 9, 9 Refused	
Does/Did he ever<br><br>(i) Force you to perform any sexual acts you did not want to?	0, 0 No | 1, 1 Yes | 8, 8 Don't know | 9, 9 Refused	
How often did this happen during the last 12 months: often, only sometimes, or not at all?	0, 0 Not at all | 1, 1 Only sometimes | 2, 2 Often | 8, 8 Don't know | 9, 9 Refused	
